# Prokaryotes Versus Eukaryotes: Who is Hosting Whom?

**DOI:** 10.3389/fvets.2014.00003

**Published:** 2014-10-14

**Authors:** Guillermo Tellez

**Affiliations:** ^1^The John Kirkpatrick Skeeles Poultry Health Laboratory, Department of Poultry Science, The Center of Excellence for Poultry Science, University of Arkansas, Fayetteville, AR, USA

**Keywords:** microbiome, prokaryotes, eukaryotes, microbial endocrinology, probiotics, digestive physiology, enteric nervous system

## Abstract

Microorganisms represent the largest component of biodiversity in our world. For millions of years, prokaryotic microorganisms have functioned as a major selective force shaping eukaryotic evolution. Microbes that live inside and on animals outnumber the animals’ actual somatic and germ cells by an estimated 10-fold. Collectively, the intestinal microbiome represents a “forgotten organ,” functioning as an organ inside another that can execute many physiological responsibilities. The nature of primitive eukaryotes was drastically changed due to the association with symbiotic prokaryotes facilitating mutual coevolution of host and microbe. Phytophagous insects have long been used to test theories of evolutionary diversification; moreover, the diversification of a number of phytophagous insect lineages has been linked to mutualisms with microbes. From termites and honey bees to ruminants and mammals, depending on novel biochemistries provided by the prokaryotic microbiome, the association helps to metabolize several nutrients that the host cannot digest and converting these into useful end products (such as short-chain fatty acids), a process, which has huge impact on the biology and homeostasis of metazoans. More importantly, in a direct and/or indirect way, the intestinal microbiota influences the assembly of gut-associated lymphoid tissue, helps to educate immune system, affects the integrity of the intestinal mucosal barrier, modulates proliferation and differentiation of its epithelial lineages, regulates angiogenesis, and modifies the activity of enteric as well as the central nervous system. Despite these important effects, the mechanisms by which the gut microbial community influences the host’s biology remain almost entirely unknown. Our aim here is to encourage empirical inquiry into the relationship between mutualism and evolutionary diversification between prokaryotes and eukaryotes, which encourage us to postulate: who is hosting whom?

## The Microbiome and General Homeostasis

A common human propensity is to regard all microorganisms as “harmful,” in particular, equating bacteria to pathogenic germs. Nothing could be further from the truth. The number of beneficial bacterial species far exceeds the number of pathogenic species and many of the known bacteria are in fact useful or even indispensable for the continued existence of life on Earth. Prokaryotic microorganisms are widespread in all environments on Earth, establishing diverse interactions with many eukaryotic taxa (1, 2). The cooperative interactions between species (mutualism) have had a central role in the generation and maintenance of life on earth (3, 4). Prokaryotes and eukaryotes are involved in diverse forms of mutualism (5). Adaptive diversification is a process intrinsically tied to species interactions (6). Yet, the influence of most types of interspecific interactions on adaptive evolutionary diversification remains poorly understood. In particular, the role of mutualistic interactions in shaping adaptive radiations has been largely unexplored, despite the ubiquity of mutualisms and increasing evidence of their ecological and evolutionary importance. The endosymbiotic theory states that several key organelles of eukaryotes originated as symbioses between separate single-celled organisms (7). According to this theory, mitochondria and plastids (e.g., chloroplasts), and possibly other organelles, represent formerly free-living bacteria that were taken inside another cell as an endosymbiont, around 1.5 billion years ago (8). Molecular and biochemical evidence suggest that the mitochondrion developed from proteobacteria and the chloroplast from cyanobacteria (9). Numerous facultative heritable endosymbionts are reproductive manipulators (5). Nevertheless, many do not manipulate reproduction, so they are expected to confer fitness benefits to their hosts, as has been shown in several studies that report defense against natural enemies, tolerance to environmental stress, and increased fecundity (6). One example of such beneficial group of microorganisms is the incredibly complex and abundant ensemble of microbes that harbors in the gastrointestinal tract (GIT) of metazoans (10). The GIT is more densely populated with microorganisms than any other organ and is an interface where the microflora may have a pronounced impact on animal biology (11–13). More than 50 genera and at least 500–1,000 different species are distributed along the length of the GIT (10, 14, 15). The bacterial population of the human cecum and colon is numerically ~10^13^ cfu/g (10, 15, 16), comprising about 40–55% of solid stool matter and weights ~1 kg (17). Presumably, the assembly of gut microflora is regulated by elaborate and combinatorial host–microbial and microbial–microbial interactions predicated on principles refined over the course of evolution (16, 18, 19). Comparison of rodents raised without exposure to any microorganisms to those colonized with an assembly of microbiota revealed a wide range of host functions affected by indigenous microbial communities. For example, the microbiota directs the assembly of the gut-associated lymphoid tissue (20, 21), helps educate the immune system (22), affects the integrity of the intestinal mucosal barrier (23), modulates proliferation and differentiation of its epithelial lineages (24), regulates angiogenesis (21), modifies the activity of the enteric nervous system (25, 26), and plays a key role in extracting and processing nutrients consumed in the diet (27, 28). The microflora can metabolize proteins and protein degradation products, sulfur-containing compounds, and endogenous and exogenous glycoproteins (29, 30). Some organisms grow on intermediate products of fermentation such as H_2_, lactate, succinate, formate, and ethanol; converting these to end products including short-chain fatty acids, a process which has direct benefits on digestive physiology (31–33). In particular with diet composition, one must conclude that, metazoans literally “become what we eat.” So, any disorders in this fragile microbial ecosystem (disbacteriosis) may predispose the host to a whole range of chronic diseases and infections thereby affecting the production of food animals. On the other hand, over millions of years, animals have developed various means for supporting complex and dynamic consortia of microorganisms during their life cycle (34, 35). As with most complex ecosystems, it appears that the majority of these microbial species cannot be cultured when removed from the niches in their host animals (24). The fragile composition of the gut microflora can be affected by various factors such as age, diet, environment, stress, and medication (36, 37). Furthermore, many factors are involved in shaping gut microflora from infancy such as mode of delivery, type of infant feeding, hospitalization, prematurity, antibiotic use, and dietary nutrient composition (36, 38). Dietary ingredients have a profound effect on the composition of the gut microflora, which in turn regulates the physiology of all animals (19). As such, nutritional components of the diet are of critical importance not only for meeting the nutrient requirements of the host but also shaping the profile of the microbiome, which in turn will determine the balance between health and disease. As an example, several studies have shown the effect of diet composition in promoting insulin sensitivity, diabetes, cancer, and other metabolic disorders (39–44). Some researchers believe that the alarming increase in autoimmune diseases in the West may owe to a disruption in the ancient relationship between our bodies and a healthy microbiome (45). Thus, colonization of microbiomes in metazoans begins at birth, and is followed by progressive assembly of a complex and dynamic microbial society maintaining a perfect harmony or homeostasis (46). However, little is known about how they influence the normal development and physiology of hosts. A transcendent view of vertebrate biology therefore requires an understanding of the contributions of these indigenous microbial communities to host development and adult physiology.

## The Microbiome and the Immune System

Today, the fields of immunology, microbiology, and nutrition converge in an astonishing way (47). Balanced gastrointestinal microflora and immune-stimulation are major functional effects attributed to beneficial bacteria (48–50). In this context, a short window of time during birth exists that enables the colonization of symbiotic bacteria to all mucosal surfaces, which may modify the future immune phenotype of the host (51–53). Perhaps, a delayed microbial colonization of the gut mucosa, the largest immune organ of the body, could cause significant changes in the immune system possibly having long term impacts on systemic immunity (54–60). For instance, some effects of the microbiome are mediated through immune regulation, particularly through balanced control of pro-inflammatory and anti-inflammatory cytokines (61, 62). Moreover, several animal and human studies have provided unequivocal evidence that specific bacterial strains are capable of stimulating multiple aspects of innate immunity (63–67) as well as to increase humoral immunity (68–70). Very interestingly, through a process of “cross talk” with the mucosal immune system, the microbiota negotiates mutual growth, survival, and inflammatory control of the intestinal ecosystem and pathogen control (71–75).

## The Microbiome–Gut–Brain Axis

Gut bacteria produce hundreds of neurochemicals that the brain uses to regulate basic physiological as well as mental processes such as learning, memory, and mood variations (76, 77). Today, it has been recognized that the microbiome–gut–brain axis influences brain chemistry and behavior independently of the autonomic nervous system, gastrointestinal-specific neurotransmitters, or inflammation (78). The ability of the microflora to synthesize neuroactive compounds such as acetylcholine, dihydroxyphenylacetic acid, l-3,4-dihydroxyphenylalanine, dopamine-4-*O*-sulfate, epinephrine, γ-aminobutyric acid, histamine, norepinephrine, serotonine, tyramine, among others, provides some microbial endocrinology-based mechanism that may help to explain some of the mechanisms, both immunological and neurophysiological components, by which the microbiome modulates the biology of their host (79). The fact that bacteria not only produce but also respond to neurochemicals produced by the host has led to the creation of a new field of study, microbial endocrinology, providing convincing evidence that cell-to-cell signaling in metazoans may be due to late horizontal gene transfer from bacteria (80). As an evidence of this astonishing link between prokaryotes and their hosts, some probiotic strains have been shown to reduce anxiety and depression by lowering the levels of corticosterone (81), as well as helping program some aspects of brain development in neonates (82–84). There is a lot of truth in the old saying “thinking with your gut,” since the enteric nervous system, known as the “second brain” contains more neurons than the peripheral nervous system (79). More recently, various studies have also linked some gut microflora with autism (85–88). Conversely, just as gut bacteria affect the brain, the brain can also exert profound influences on the gut microbiome, with feedback effects on behavior. This may help explain why people with inflammatory syndromes such as Crohn’s disease, ulcerative colitis, or irritable bowel syndrome are also plagued by anxiety and depression (89–91). High and chronic levels of stress hormones such as epinephrine and norepinephrine have been shown to increase virulence and pathogenicity of several pathogenic enterobacteria (92–95). This neurochemical-mediated “two-way street” is one of the principles that undergirds the microbial endocrinology construct that unify animal and plant kingdoms with the same origin, since for example, stress-related neuroendocrine hormone family of catecholamines has also been demonstrated in all forms of life on earth (79).

## Who is Hosting Whom?

*Wolbachia* are common intracellular bacteria that are found in arthropods and nematodes (5). It is one of the world’s most common parasitic microbes and is possibly the most common reproductive parasite in the biosphere (96). Its interactions with its hosts are often complex, and in some cases have evolved to be mutualistic rather than parasitic. Some host species cannot reproduce, or even survive, without *Wolbachia* infection (97). These proteobacteria endosymbionts are transmitted vertically through host eggs and alter host biology in diverse ways, including the induction of reproductive manipulations, such as feminization, parthenogenesis, male killing, and sperm-egg incompatibility and they can also move horizontally across species boundaries, resulting in a widespread and global distribution in diverse invertebrate hosts (98).

Prokaryotes also have developed a sophisticated system of stimulus, communication, and response (*quorum sensing*) that many species of bacteria use to coordinate gene expression that coordinate certain behaviors such as biofilm formation, virulence, and antibiotic resistance, based on the local density of the bacterial population (99–102). *Quorum sensing* occurs within a single bacterial species as well as between diverse species, and the cells of the host, serving as a simple indicator of population density or the diffusion rate of the cell’s immediate environment (103–107).

At a higher biological level, there is clear evidence that even some parasites can be also excellent manipulators of their host. It is remarkable that when two parasite species are manipulators and have different definitive hosts, there is a potential for conflict between them. Selection may then exist for either avoiding hosts infected with conflicting parasites, or for hijacking, i.e., competitive processes to gain control of the intermediate host (108). The evidence for both phenomena depends largely on the study of the relative competitive abilities of parasites within their common intermediate host. Adaptive host manipulation hypothesis is usually supported by case studies on trophically transmitted heteroxenous endoparasites. Trematodes and cestodes are among efficient manipulators of fish, their common intermediate hosts. Reviewed experimental data suggest that heteroxenous parasites manipulate their host mainly through impaired defense behavior, e.g., impairing shoaling in fish. Alternatively, monoxenous parasites facilitate shoaling that is profitable for both parasites and hosts (109). Coordination of modified host behavior with the parasite life cycle, both temporal and spatial, is the most convincing criterion of the adaptive value of host manipulation (110). In human beings, seropositivity of the obligate intracellular protozoan parasite, *T. gondii* is related to various mental health disorders including schizophrenia, suicide attempt, depression, and other neuropsychiatric diseases (111). Depressive symptoms have been linked to interferon-γ (IFN-γ) blocking *T. gondii* growth by inducing indoleamine-2,3-dioxygenase (IDO) activation and tryptophan depletion, which results in a decrease of serotonin production in the brain (112).

## Final Remarks

The interest in digestive physiology and the role of microorganisms has generated data whereby human and animal well being can be enhanced and the risk of disease reduced. New molecular techniques that allow more accurate assessment of the flora composition are resulting in improved strategies for elucidating mechanisms. The research field of beneficial microorganisms to animals and humans (probiotics) has been aimed at modulating the intestinal microflora and current research is still heavily biased toward gastrointestinal applications for probiotics, such as chronic constipation (113), chronic diarrhea (114), inflammatory bowel disease (115), irritable bowel syndrome (116), and food allergy (117), the possibilities for impacting many areas of health are numerous. However, other parts of the body containing endogenous microflora or problems relating to the immune system may also be candidates for probiotic therapy. A few researchers have already shown that probiotics have potential for human health issues such as vaginal candidiosis (118), dental caries (119), allergies (120), autoimmune diseases (121), urogenital infections (122), atopic diseases (69), rheumatoid arthritis (26), and respiratory infections (68). It has been said that “nothing is new under the sun.” There are no new or novel discoveries in this review that have not been clearly described previously by numerous scientists. Rather, the purpose of this manuscript is simply trying to put some of the puzzle pieces together. When we ponder and contemplate the astonishing and remarkable roles that prokaryotes have on host metabolism, immune function, gene expression, reproduction, and behavior of metazoans, I wonder who is, in reality, hosting whom?

## Conflict of Interest Statement

The author declares that the research was conducted in the absence of any commercial or financial relationships that could be construed as a potential conflict of interest.
